# Complete genome sequence of *Virgibacillus* sp. KFRI-KCUT25010 isolated from a fermented hairtail (*Trichiurus lepturus*) sauce

**DOI:** 10.1128/mra.01489-25

**Published:** 2026-03-13

**Authors:** Myunglip Lee, Yukyoung Park, Sunghun Yi

**Affiliations:** 1 Fermentation Convergence Research Group, Korea Food Research Institute (KFRI), Wanju-gun, Jeollabuk-do, Republic of Korea; Nanchang University, Nanchang, Jiangxi, China

**Keywords:** *Virgibacillus*, genome, PacBio Revio, Illumina NovaSeq

## Abstract

*Virgibacillus* sp. strain KFRI-KCUT25010 was isolated from fermented hairtail sauce in Korea and sequenced using Illumina NovaSeq X and PacBio Revio HiFi technologies. The genome comprises a single 3.78 Mb circular chromosome with 39.6% GC content. Comparative analyses place the strain within *Virgibacillus* as a genomically distinct lineage based on ANI.

## ANNOUNCEMENT

The genus *Virgibacillus* (family *Bacillaceae*) comprises moderately halophilic, endospore-forming bacteria frequently isolated from saline environments and fermented foods, including fermented seafood as an important ecological niche for discovering novel halophilic taxa (1–5). *Virgibacillus* sp. strain KFRI-KCUT25010 was obtained from fermented hairtail (*Trichiurus lepturus*) sauce (galchi-jeotgal) purchased at a local market in Gang-gyeong, Korea in 2024 (36.15° N, 127.01° E). A 20-g sample was homogenized with 180 mL of sterile 0.85% (w/v) NaCl using a stomacher homogenizer, serially diluted (10^−3^ to 10^−5^) and spread-plated onto Marine Agar 2216 (Difco). Plates were incubated aerobically at 30°C for 72 h. A single colony was purified and preserved at −80°C in 20% (v/v) glycerol stocks. Genomic DNA was extracted using the QIAamp DNA Mini Kit (QIAGEN) according to the manufacturer’s protocol.

Whole-genome sequencing used a hybrid approach combining Illumina NovaSeq X short-read libraries with PacBio Revio long-read libraries (HiFi CCS reads). Illumina libraries were prepared using the Nextera DNA Flex Library Prep Kit (Illumina) following the manufacturer’s protocol without modification. Illumina sequencing was performed on a NovaSeq X platform to generate 2 × 151 bp paired-end reads. PacBio libraries were prepared using the SMRTbell Express Template Prep Kit v2.0 without DNA shearing or size selection, sequenced on the PacBio Revio platform using HiFi CCS chemistry v3.0. Read quality was assessed with FastQC v0.11.7 (http://www.bioinformatics.babraham.ac.uk/projects/fastqc). Illumina adapters and low-quality bases were removed with Trimmomatic v0.38 (6). Jellyfish v1.1.12 was used for k-mer counting. Long reads were assembled with Canu v1.7, and assemblies were polished with Pilon v1.22 and refined in a hybrid manner using the Microbial Genome Analysis pipeline (SMRT Link v13.1.0.221970) (7, 8). PacBio HiFi reads were aligned and the assembly validated with pbmm2 v12.1 (https://github.com/PacificBiosciences/pbmm2). Genome completeness was evaluated using BUSCO v5.1.3 with the bacteria_odb10 data set (9). Structural and functional annotations were generated using NCBI PGAP and Prokka v1.14.6, with functional assignments complemented by eggNOG v4.5 (10, 11). For the circular genome, terminal overlap was identified during assembly, trimmed to generate a single circular contig, and the chromosome was rotated to start at the *dnaA* gene. Default parameters were used except where otherwise noted. Pairwise 16S rRNA gene identity, ANI, and AAI were calculated as described previously (12–14).

Illumina NovaSeq X produced 22,270,204 paired reads (3.36 Gb; Q30 95.7%), and PacBio Revio HiFi yielded 83,782 circular consensus reads (625.7 Mb; read N50 8,775 bp). *De novo* assembly yielded a single circular chromosome, and detailed genome statistics are summarized in Table 1. Assembly completeness was 99.2% based on BUSCO analysis, indicating a near-complete genome. Genome assembly and annotation statistics are summarized in Table 1. We combined 16S rRNA gene comparisons with whole-genome relatedness analyses. 16S rRNA genes were retrieved from the assembly. EzBioCloud-based 16S rRNA gene identification indicated the closest validly published type strain is *Virgibacillus natechei* FarDᵀ (NCBI assembly ASM2601364v1), showing 96.91% sequence similarity. Comparative genomic analysis revealed that strain KFRI-KCUT25010 shared average nucleotide identity (ANI) values of 79.12% and average amino acid identity (AAI) values of 79.99%. These values are substantially below the commonly used species delineation threshold of 95%–96%. Collectively, these results indicate that strain KFRI-KCUT25010 represents a genomically distinct lineage within the genus *Virgibacillus* (15).

**TABLE 1:** Genome assembly and annotation statistics for *Virgibacillus* sp. KCUT25010

Feature	Value
Genome size (bp)	3,776,343
GC content (%)	39.6
Contigs (topology)	1^a^
Mean coverage (×)	166
Contig N50 (bp)	3,776,343
Total genes	3,851
Protein-coding genes (CDS)	3,765
rRNA	6, 6, 6 (5S, 16S, 23S)
tRNA / ncRNA	63 / 5
BUSCO completeness (%)	99.2

^a^ Single circular chromosome.

## Data Availability

The complete genome sequence of *Virgibacillus* sp. KFRI-KCUT25010 has been deposited in GenBank under accession CP199871. The BioProject accession is PRJNA1321406, and raw Illumina and PacBio reads will be available in the NCBI SRA under accession numbers SRX30398050 and SRX30398051.
